# The Effects of Turbulent Biological Tissue on Adjustable Anomalous Vortex Laser Beam

**DOI:** 10.3390/biomimetics10070461

**Published:** 2025-07-14

**Authors:** Yiqun Zhang, Wu Wang, Xiaokun Ding, Liyu Sun, Zhenyang Qian, Huilin Jiang, Yansong Song, Runwei Ding

**Affiliations:** 1 School of Optoelectronic Engineering, Changchun University of Science and Technology, Changchun 130013, China; zhangyq@pcl.ac.cn (Y.Z.);; 2 Peng Cheng Laboratory, Shenzhen 518055, China; 3 North Navigation Control Technology Co., Ltd., Beijing 100072, China; 4 AVIC Flight Automatic Control Research Institute, Northwestern Polytechnical University, Xi’an 710072, China; 5 AVIC Flight Automatic Control Research Institute, Xi’an 710065, China

**Keywords:** laser, interdisciplinary research, intensity, biological tissue

## Abstract

In this work, we present a new partially coherent adjustable anomalous vortex laser beam (PCAAVLB) and introduce it into turbulent biological tissue. The equation of such PCAAVLB in turbulent biological tissue is obtained. By numerical analysis, the evolution of the intensity of such PCAAVLB in turbulent biological tissue is analyzed. It is found that the PCAAVLB in biological tissue can lose its ring shape and become a Gaussian beam, and a PCAAVLB with smaller topological charge M or coherence length σ will evolve into a Gaussian profile faster. The PCAAVLB in turbulent biological tissue with a smaller small-length-scale factor $l_{0}$ or larger fractal dimension D will evolve into a Gaussian profile faster and have a larger intensity as z increases. The results may have potential applications in sensing under biological tissue environments and laser imaging in biology.

## 1. Introduction

The properties of tissue affect the application of lasers in medicine, and the optical properties of tissue have been investigated [1]. To explore the applications of lasers in biological tissues, the influences of tissue turbulence on optical waves have attracted attention [2], as have the properties (intensity and beam spread et al.) of light in biological tissues. In addition, the intensity of various lasers in turbulent biological tissue has been studied, such as the hollow Gaussian beam [3], partially coherent beam [4], vortex beam [5], Hermite–Gaussian correlated beam [6], rectangular multi-Gaussian beam [7], and Gaussian–Schell model vortex beam [8]. The coherence properties of laser in tissue are still analyzed [9]. Moreover, the coupling efficiency to fiber [10] and adaptive optics correction in tissues [11] are also investigated. From the above references, one sees that different types of laser beams may have potential applications in tissues.

A vortex is a signature of natural systems and is observed in fluids, smoke rings, and tornados [12]. The high speed of the peregrine falcon can be enhanced by the vortices emanating from the frontal and dorsal regions [13]. The trailing-edge vortices have been found in the flight of mosquitoes [14]. Moreover, the vortex has also been introduced into the light, and the double vortex beams have also been investigated [15,16]. The intensity pattern of a laser usually has a Gaussian profile. However, the intensity pattern of light can be modulated, and an anomalous hollow beam can show a unique intensity profile [17]. And the propagation of such AHB has been studied [18,19,20]. Such an AHB has also been extended into a partially coherent beam [21,22,23]. Recently, a new adjustable anomalous vortex beam (AAVB) has been introduced [24]; the properties of such AAVB can be modulated by adjustable parameters. It will be interesting if this adjustable beam is introduced into biological tissue.

The vortex in nature [12], birds [13] and mosquitoes [14], shows unique properties. In this work, the AAVB is extended into a partially coherent beam, and a new partially coherent adjustable anomalous vortex laser beam (PCAAVLB), which can be modulated by the adjustable parameters, is introduced. The intensity of the PCAAVLB can be controlled by adjustable parameters, while the intensity of the multi-Gaussian correlated AHB has the ring symmetry in Reference [23]. This PCAAVLB is introduced into the tissues, and the intensity equation of such PCAAVLB in turbulent biological tissue is derived. Based on the derived equations, the effects of turbulent biological tissue on the intensity of such PCAAVLB are analyzed.

## 2. Propagation of PCAAVLB in Turbulent Biological Tissue

When the PCAAVLB propagates along the z-axis, the intensity of PCAAVLB in turbulent biological tissue is described by the Huygens–Fresnel integral as follows [3,4,5,6,7,8]:

(1) $$\begin{array}{l} I\left(\rho ,z\right)=\frac{k^{2}}{4\pi^{2}z^{2}}\int dr_{1}dr_{2}W_{0}\left(r_{1},r_{2}\right)\\ \times \exp\left[-\frac{ik}{2z}{\left(\rho -r_{1}\right)}^{2}+\frac{ik}{2z}{\left(\rho -r_{2}\right)}^{2}\right]\langle \exp\left[\psi \left(r_{1},\rho \right)+\psi^{*}\left(r_{2},\rho \right)\right]\rangle \end{array}$$

where $I\left(\rho ,z\right)$ represents the intensity; $W_{0}\left(r_{1},r_{2}\right)$ is the cross spectral density (CSD) of laser at z=0; k=2π/λ is the wavenumber with λ is the wavelength in vacuum; $r=\left(x,y\right)$ and $\rho =\left(\rho_{x},\rho_{y}\right)$ are position vectors at z=0 and z, respectively; i is imaginary unit; $\psi \left(r,\rho \right)$ is the phase perturbation of biological tissue.

(2) $$\langle \exp\left[\psi \left(r_{1},\rho \right)+\psi^{*}\left(r_{2},\rho \right)\right]\rangle =\exp\left[-\frac{{\left(x_{1}-x_{2}\right)}^{2}+{\left(y_{1}-y_{2}\right)}^{2}}{\Lambda^{2}}\right]$$

The Λ is the coherence length of a spherical wave in turbulent biological tissue and which can be written as follows [10]:

(3) $$\Lambda ={\left[\frac{\pi^{1/2}k^{2}Sz}{3\times 2^{\left(7-D\right)/2}l_{c}}\Gamma \left(\frac{D}{2}\right)U\left(2;\frac{7}{6};\frac{l_{0}^{2}}{8\ln\left(2\right)l_{c}^{2}}\right)\right]}^{-1/2}$$

where S denotes the strength coefficient of biological tissues; D denotes the fractal dimension; $l_{c}$ denotes the characteristic length of heterogeneity; $l_{0}$ denotes small length-scale factor; $\Gamma \left(\cdot \right)$ denotes the gamma function; $U\left(\cdot \right)$ denotes the confluent hypergeometric function of the second kind.

The electric field of AAVB at z=0 is written as follows [24,25]:

(4) $$E\left(r,0\right)=\left(-a+c_{x}\frac{x^{2}}{w_{x}^{2}}+c_{y}\frac{y^{2}}{w_{y}^{2}}\right)\exp\left(-\frac{x^{2}}{w_{x}^{2}}-\frac{y^{2}}{w_{y}^{2}}\right){\left(x+iy\right)}^{M}$$

where $w_{x}$ and $w_{y}$ are the beam width of the Gaussian part; M is a topological charge; and a, $c_{x}$, and $c_{y}$ are adjustable parameters.

Considering the coherence theory [26], the CSD of a PCAAVLB is written as follows:

(5) $$\begin{array}{c} W_{0}\left(r_{1},r_{2}\right)=\left(-a+c_{x}\frac{{x_{1}}^{2}}{w_{x}^{2}}+c_{y}\frac{{y_{1}}^{2}}{w_{y}^{2}}\right)\exp\left(-\frac{{x_{1}}^{2}}{w_{x}^{2}}-\frac{{y_{1}}^{2}}{w_{y}^{2}}\right){\left(x_{1}+iy_{1}\right)}^{M}\\ \times \left(-a+c_{x}\frac{{x_{2}}^{2}}{w_{x}^{2}}+c_{y}\frac{{y_{2}}^{2}}{w_{y}^{2}}\right)\exp\left(-\frac{{x_{2}}^{2}}{w_{x}^{2}}-,\frac{{y_{2}}^{2}}{w_{y}^{2}}\right){\left(x_{2}+{iy}_{2}\right)}^{M}\\ \times \exp\left[-\frac{{\left(x_{1}-x_{2}\right)}^{2}}{2\sigma_{x}^{2}}-\frac{{\left(y_{1}-y_{2}\right)}^{2}}{2\sigma_{y}^{2}}\right] \end{array}$$

where $\sigma_{x}$ and $\sigma_{y}$ are the coherence length along x-axis and y-axis. The intensity of PCAAVLB can be modulated by the adjustable parameters in Equation (5), and this beam is newly introduced, which is different from the multi-Gaussian correlated partially coherent anomalous hollow beam in Reference [23].

We next consider the following equations [27]:

(6) $${\left(x+iy\right)}^{M}=\sum_{l=0}^{M}\frac{M!i^{l}}{l!\left(M-l\right)!}x^{M-1}y^{l}$$

(7) $$\int_{-\infty }^{+\infty }x^{n}\exp\left(-px^{2}+2qx\right)dx=n!\exp\left(\frac{q^{2}}{p}\right){\left(\frac{q}{p}\right)}^{n}\sqrt{\frac{\pi }{p}}\sum_{l=0}^{\left[\frac{n}{2}\right]}\frac{1}{l!\left(n-2l\right)!}{\left(\frac{p}{4q^{2}}\right)}^{l}$$

By substituting Equation (5) into Equation (1), the intensity of such PCAAVLB in turbulent biological tissue is derived as follows:

(8) $$\begin{array}{l} I\left(\rho ,z\right)=\frac{k^{2}}{4\pi^{2}z^{2}}\sum_{m=0}^{M}\frac{M!i^{m}}{m!\left(M-m\right)!}\sum_{l=0}^{M}\frac{M!i^{n}}{n!\left(M-n\right)!}\\ \left(a^{2}I_{1}-\frac{ac_{x}}{w_{x}^{2}}I_{2}-\frac{ac_{y}}{w_{y}^{2}}I_{3}-\frac{ac_{x}}{w_{x}^{2}}I_{4}-\frac{ac_{y}}{w_{y}^{2}}I_{5}+\frac{c_{x}^{2}}{w_{x}^{4}}I_{6}+\frac{c_{x}c_{y}}{w_{x}^{2}w_{y}^{2}}I_{7}+\frac{c_{x}c_{y}}{w_{x}^{2}w_{y}^{2}}I_{8}+\frac{c_{y}^{2}}{w_{y}^{4}}I_{9}\right) \end{array}$$

with

(9) $$\begin{array}{l} I_{1}=\sqrt{\frac{\pi }{a_{x}}}\left(M-m\right)!{\left(\frac{1}{a_{x}}\right)}^{M-m}\exp\left[\frac{1}{a_{x}}{\left(\frac{ik}{2z}\rho_{x}\right)}^{2}\right]\\ \sum_{k=0}^{\left[\frac{M-m}{2}\right]}\frac{1}{k!\left(M-m-2k\right)!}{\left(\frac{a_{x}}{4}\right)}^{k}\sum_{l=0}^{\left[M-m-2k\right]}\frac{\left(M-m-2k\right)!}{l!\left(M-m-2k-l\right)!}{\left(\frac{ik}{2z}\rho_{x}\right)}^{M-m-2k-l}\\ {\left(\frac{1}{2\sigma_{x}^{2}}+\frac{1}{\Lambda^{2}}\right)}^{l}\sqrt{\frac{\pi }{b_{x}}}2^{-\left(M-n+l\right)}i^{M-n+l}\exp\left(\frac{c_{x}^{2}}{b_{x}}\right){\left(\frac{1}{b_{x}}\right)}^{0.5\left(M-n+l\right)}H_{M-n+l}\left(\frac{ic_{x}}{\sqrt{b_{x}}}\right)\\ \sqrt{\frac{\pi }{a_{y}}}m!{\left(\frac{1}{a_{y}}\right)}^{m}\exp\left[\frac{1}{a_{y}}{\left(\frac{ik}{2z}\rho_{y}\right)}^{2}\right]\\ \sum_{t=0}^{\left[\frac{m}{2}\right]}\frac{1}{t!\left(m-2t\right)!}{\left(\frac{a_{y}}{4}\right)}^{t}\sum_{l\prime =0}^{\left[m-2t\right]}\frac{\left(m-2t\right)!}{l\prime !\left(m-2t-l\prime \right)!}{\left(\frac{ik}{2z}\rho_{y}\right)}^{m-2t-l\prime }{\left(\frac{1}{2\sigma_{y}^{2}}+\frac{1}{\Lambda^{2}}\right)}^{l\prime }\\ \sqrt{\frac{\pi }{b_{y}}}2^{-\left(n+l\prime \right)}i^{n+l\prime }\exp\left(\frac{c_{y}^{2}}{b_{y}}\right){\left(\frac{1}{b_{y}}\right)}^{0.5\left(n+l\prime \right)}H_{n+l\prime }\left(\frac{ic_{y}}{\sqrt{b_{y}}}\right) \end{array}$$

(10) $$\begin{array}{l} I_{2}=\sqrt{\frac{\pi }{a_{x}}}\left(M-m+2\right)!{\left(\frac{1}{a_{x}}\right)}^{M-m+2}\exp\left[\frac{1}{a_{x}}{\left(\frac{ik}{2z}\rho_{x}\right)}^{2}\right]\\ \sum_{k=0}^{\left[\frac{M-m+2}{2}\right]}\frac{1}{k!\left(M-m+2-2k\right)!}{\left(\frac{a_{x}}{4}\right)}^{k}\sum_{l=0}^{\left[M-m+2-2k\right]}\frac{\left(M-m+2-2k\right)!}{l!\left(M-m+2-2k-l\right)!}{\left(\frac{ik}{2z}\rho_{x}\right)}^{M-m+2-2k-l}\\ {\left(\frac{1}{2\sigma_{x}^{2}}+\frac{1}{\Lambda^{2}}\right)}^{l}\sqrt{\frac{\pi }{b_{x}}}2^{-\left(M-n+l\right)}i^{M-n+l}\exp\left(\frac{c_{x}^{2}}{b_{x}}\right){\left(\frac{1}{b_{x}}\right)}^{0.5\left(M-n+l\right)}H_{M-n+l}\left(\frac{ic_{x}}{\sqrt{b_{x}}}\right)\\ \sqrt{\frac{\pi }{a_{y}}}m!{\left(\frac{1}{a_{y}}\right)}^{m}\exp\left[\frac{1}{a_{y}}{\left(\frac{ik}{2z}\rho_{y}\right)}^{2}\right]\\ \sum_{t=0}^{\left[\frac{m}{2}\right]}\frac{1}{t!\left(m-2t\right)!}{\left(\frac{a_{y}}{4}\right)}^{t}\sum_{l\prime =0}^{\left[m-2t\right]}\frac{\left(m-2t\right)!}{l\prime !\left(m-2t-l\prime \right)!}{\left(\frac{ik}{2z}\rho_{y}\right)}^{m-2t-l\prime }{\left(\frac{1}{2\sigma_{y}^{2}}+\frac{1}{\Lambda^{2}}\right)}^{l\prime }\\ \sqrt{\frac{\pi }{b_{y}}}2^{-\left(n+l\prime \right)}i^{n+l\prime }\exp\left(\frac{c_{y}^{2}}{b_{y}}\right){\left(\frac{1}{b_{y}}\right)}^{0.5\left(n+l\prime \right)}H_{n+l\prime }\left(\frac{ic_{y}}{\sqrt{b_{y}}}\right) \end{array}$$

(11) $$\begin{array}{l} I_{3}=\sqrt{\frac{\pi }{a_{x}}}\left(M-m\right)!{\left(\frac{1}{a_{x}}\right)}^{M-m}\exp\left[\frac{1}{a_{x}}{\left(\frac{ik}{2z}\rho_{x}\right)}^{2}\right]\\ \sum_{k=0}^{\left[\frac{M-m}{2}\right]}\frac{1}{k!\left(M-m-2k\right)!}{\left(\frac{a_{x}}{4}\right)}^{k}\sum_{l=0}^{\left[M-m-2k\right]}\frac{\left(M-m-2k\right)!}{l!\left(M-m-2k-l\right)!}{\left(\frac{ik}{2z}\rho_{x}\right)}^{M-m-2k-l}\\ {\left(\frac{1}{2\sigma_{x}^{2}}+\frac{1}{\Lambda^{2}}\right)}^{l}\sqrt{\frac{\pi }{b_{x}}}2^{-\left(M-n+l\right)}i^{M-n+l}\exp\left(\frac{c_{x}^{2}}{b_{x}}\right){\left(\frac{1}{b_{x}}\right)}^{0.5\left(M-n+l\right)}H_{M-n+l}\left(\frac{ic_{x}}{\sqrt{b_{x}}}\right)\\ \sqrt{\frac{\pi }{a_{y}}}\left(m+2\right)!{\left(\frac{1}{a_{y}}\right)}^{m+2}\exp\left[\frac{1}{a_{y}}{\left(\frac{ik}{2z}\rho_{y}\right)}^{2}\right]\\ \sum_{t=0}^{\left[\frac{m+2}{2}\right]}\frac{1}{t!\left(m+2-2t\right)!}{\left(\frac{a_{y}}{4}\right)}^{t}\sum_{l\prime =0}^{\left[m+2-2t\right]}\frac{\left(m+2-2t\right)!}{l\prime !\left(m+2-2t-l\prime \right)!}{\left(\frac{ik}{2z}\rho_{y}\right)}^{m+2-2t-l\prime }{\left(\frac{1}{2\sigma_{y}^{2}}+\frac{1}{\Lambda^{2}}\right)}^{l\prime }\\ \sqrt{\frac{\pi }{b_{y}}}2^{-\left(n+l\prime \right)}i^{n+l\prime }\exp\left(\frac{c_{y}^{2}}{b_{y}}\right){\left(\frac{1}{b_{y}}\right)}^{0.5\left(n+l\prime \right)}H_{n+l\prime }\left(\frac{ic_{y}}{\sqrt{b_{y}}}\right) \end{array}$$

(12) $$\begin{array}{l} I_{4}=\sqrt{\frac{\pi }{a_{x}}}\left(M-m\right)!{\left(\frac{1}{a_{x}}\right)}^{M-m}\exp\left[\frac{1}{a_{x}}{\left(\frac{ik}{2z}\rho_{x}\right)}^{2}\right]\\ \sum_{k=0}^{\left[\frac{M-m}{2}\right]}\frac{1}{k!\left(M-m-2k\right)!}{\left(\frac{a_{x}}{4}\right)}^{k}\sum_{l=0}^{\left[M-m-2k\right]}\frac{\left(M-m-2k\right)!}{l!\left(M-m-2k-l\right)!}{\left(\frac{ik}{2z}\rho_{x}\right)}^{M-m-2k-l}\\ {\left(\frac{1}{2\sigma_{x}^{2}}+\frac{1}{\Lambda^{2}}\right)}^{l}\sqrt{\frac{\pi }{b_{x}}}2^{-\left(M-n+2+l\right)}i^{M-n+2+l}\exp\left(\frac{c_{x}^{2}}{b_{x}}\right){\left(\frac{1}{b_{x}}\right)}^{0.5\left(M-n+2+l\right)}H_{M-n+2+l}\left(\frac{ic_{x}}{\sqrt{b_{x}}}\right)\\ \sqrt{\frac{\pi }{a_{y}}}m!{\left(\frac{1}{a_{y}}\right)}^{m}\exp\left[\frac{1}{a_{y}}{\left(\frac{ik}{2z}\rho_{y}\right)}^{2}\right]\\ \sum_{t=0}^{\left[\frac{m}{2}\right]}\frac{1}{t!\left(m-2t\right)!}{\left(\frac{a_{y}}{4}\right)}^{t}\sum_{l\prime =0}^{\left[m-2t\right]}\frac{\left(m-2t\right)!}{l\prime !\left(m-2t-l\prime \right)!}{\left(\frac{ik}{2z}\rho_{y}\right)}^{m-2t-l\prime }{\left(\frac{1}{2\sigma_{y}^{2}}+\frac{1}{\Lambda^{2}}\right)}^{l\prime }\\ \sqrt{\frac{\pi }{b_{y}}}2^{-\left(n+l\prime \right)}i^{n+l\prime }\exp\left(\frac{c_{y}^{2}}{b_{y}}\right){\left(\frac{1}{b_{y}}\right)}^{0.5\left(n+l\prime \right)}H_{n+l\prime }\left(\frac{ic_{y}}{\sqrt{b_{y}}}\right) \end{array}$$

(13) $$\begin{array}{l} I_{5}=\sqrt{\frac{\pi }{a_{x}}}\left(M-m\right)!{\left(\frac{1}{a_{x}}\right)}^{M-m}\exp\left[\frac{1}{a_{x}}{\left(\frac{ik}{2z}\rho_{x}\right)}^{2}\right]\\ \sum_{k=0}^{\left[\frac{M-m}{2}\right]}\frac{1}{k!\left(M-m-2k\right)!}{\left(\frac{a_{x}}{4}\right)}^{k}\sum_{l=0}^{\left[M-m-2k\right]}\frac{\left(M-m-2k\right)!}{l!\left(M-m-2k-l\right)!}{\left(\frac{ik}{2z}\rho_{x}\right)}^{M-m-2k-l}\\ {\left(\frac{1}{2\sigma_{x}^{2}}+\frac{1}{\Lambda^{2}}\right)}^{l}\sqrt{\frac{\pi }{b_{x}}}2^{-\left(M-n+l\right)}i^{M-n+l}\exp\left(\frac{c_{x}^{2}}{b_{x}}\right){\left(\frac{1}{b_{x}}\right)}^{0.5\left(M-n+l\right)}H_{M-n+l}\left(\frac{ic_{x}}{\sqrt{b_{x}}}\right)\\ \sqrt{\frac{\pi }{a_{y}}}m!{\left(\frac{1}{a_{y}}\right)}^{m}\exp\left[\frac{1}{a_{y}}{\left(\frac{ik}{2z}\rho_{y}\right)}^{2}\right]\\ \sum_{t=0}^{\left[\frac{m}{2}\right]}\frac{1}{t!\left(m-2t\right)!}{\left(\frac{a_{y}}{4}\right)}^{t}\sum_{l\prime =0}^{\left[m-2t\right]}\frac{\left(m-2t\right)!}{l\prime !\left(m-2t-l\prime \right)!}{\left(\frac{ik}{2z}\rho_{y}\right)}^{m-2t-l\prime }{\left(\frac{1}{2\sigma_{y}^{2}}+\frac{1}{\Lambda^{2}}\right)}^{l\prime }\\ \sqrt{\frac{\pi }{b_{y}}}2^{-\left(n+2+l\prime \right)}i^{n+2+l\prime }\exp\left(\frac{c_{y}^{2}}{b_{y}}\right){\left(\frac{1}{b_{y}}\right)}^{0.5\left(n+2+l\prime \right)}H_{n+l\prime }\left(\frac{ic_{y}}{\sqrt{b_{y}}}\right) \end{array}$$

(14) $$\begin{array}{l} I_{6}=\sqrt{\frac{\pi }{a_{x}}}\left(M-m+2\right)!{\left(\frac{1}{a_{x}}\right)}^{M-m+2}\exp\left[\frac{1}{a_{x}}{\left(\frac{ik}{2z}\rho_{x}\right)}^{2}\right]\\ \sum_{k=0}^{\left[\frac{M-m+2}{2}\right]}\frac{1}{k!\left(M-m+2-2k\right)!}{\left(\frac{a_{x}}{4}\right)}^{k}\sum_{l=0}^{\left[M-m+2-2k\right]}\frac{\left(M-m+2-2k\right)!}{l!\left(M-m+2-2k-l\right)!}{\left(\frac{ik}{2z}\rho_{x}\right)}^{M-m+2-2k-l}\\ {\left(\frac{1}{2\sigma_{x}^{2}}+\frac{1}{\Lambda^{2}}\right)}^{l}\sqrt{\frac{\pi }{b_{x}}}2^{-\left(M-n+2+l\right)}i^{M-n+2+l}\exp\left(\frac{c_{x}^{2}}{b_{x}}\right){\left(\frac{1}{b_{x}}\right)}^{0.5\left(M-n+2+l\right)}H_{M-n+2+l}\left(\frac{ic_{x}}{\sqrt{b_{x}}}\right)\\ \sqrt{\frac{\pi }{a_{y}}}m!{\left(\frac{1}{a_{y}}\right)}^{m}\exp\left[\frac{1}{a_{y}}{\left(\frac{ik}{2z}\rho_{y}\right)}^{2}\right]\\ \sum_{t=0}^{\left[\frac{m}{2}\right]}\frac{1}{t!\left(m-2t\right)!}{\left(\frac{a_{y}}{4}\right)}^{t}\sum_{l\prime =0}^{\left[m-2t\right]}\frac{\left(m-2t\right)!}{l\prime !\left(m-2t-l\prime \right)!}{\left(\frac{ik}{2z}\rho_{y}\right)}^{m-2t-l\prime }{\left(\frac{1}{2\sigma_{y}^{2}}+\frac{1}{\Lambda^{2}}\right)}^{l\prime }\\ \sqrt{\frac{\pi }{b_{y}}}2^{-\left(n+l\prime \right)}i^{n+l\prime }\exp\left(\frac{c_{y}^{2}}{b_{y}}\right){\left(\frac{1}{b_{y}}\right)}^{0.5\left(n+l\prime \right)}H_{n+l\prime }\left(\frac{ic_{y}}{\sqrt{b_{y}}}\right) \end{array}$$

(15) $$\begin{array}{l} I_{7}=\sqrt{\frac{\pi }{a_{x}}}\left(M-m+2\right)!{\left(\frac{1}{a_{x}}\right)}^{M-m+2}\exp\left[\frac{1}{a_{x}}{\left(\frac{ik}{2z}\rho_{x}\right)}^{2}\right]\\ \sum_{k=0}^{\left[\frac{M-m+2}{2}\right]}\frac{1}{k!\left(M-m+2-2k\right)!}{\left(\frac{a_{x}}{4}\right)}^{k}\sum_{l=0}^{\left[M-m+2-2k\right]}\frac{\left(M-m+2-2k\right)!}{l!\left(M-m+2-2k-l\right)!}{\left(\frac{ik}{2z}\rho_{x}\right)}^{M-m+2-2k-l}\\ {\left(\frac{1}{2\sigma_{x}^{2}}+\frac{1}{\Lambda^{2}}\right)}^{l}\sqrt{\frac{\pi }{b_{x}}}2^{-\left(M-n+l\right)}i^{M-n+l}\exp\left(\frac{c_{x}^{2}}{b_{x}}\right){\left(\frac{1}{b_{x}}\right)}^{0.5\left(M-n+l\right)}H_{M-n+l}\left(\frac{ic_{x}}{\sqrt{b_{x}}}\right)\\ \sqrt{\frac{\pi }{a_{y}}}m!{\left(\frac{1}{a_{y}}\right)}^{m}\exp\left[\frac{1}{a_{y}}{\left(\frac{ik}{2z}\rho_{y}\right)}^{2}\right]\\ \sum_{t=0}^{\left[\frac{m}{2}\right]}\frac{1}{t!\left(m-2t\right)!}{\left(\frac{a_{y}}{4}\right)}^{t}\sum_{l\prime =0}^{\left[m-2t\right]}\frac{\left(m-2t\right)!}{l\prime !\left(m-2t-l\prime \right)!}{\left(\frac{ik}{2z}\rho_{y}\right)}^{m-2t-l\prime }{\left(\frac{1}{2\sigma_{y}^{2}}+\frac{1}{\Lambda^{2}}\right)}^{l\prime }\\ \sqrt{\frac{\pi }{b_{y}}}2^{-\left(n+2+l\prime \right)}i^{n+l\prime }\exp\left(\frac{c_{y}^{2}}{b_{y}}\right){\left(\frac{1}{b_{y}}\right)}^{0.5\left(n+2+l\prime \right)}H_{n+l\prime }\left(\frac{ic_{y}}{\sqrt{b_{y}}}\right) \end{array}$$

(16) $$\begin{array}{l} I_{8}=\sqrt{\frac{\pi }{a_{x}}}\left(M-m\right)!{\left(\frac{1}{a_{x}}\right)}^{M-m}\exp\left[\frac{1}{a_{x}}{\left(\frac{ik}{2z}\rho_{x}\right)}^{2}\right]\\ \sum_{k=0}^{\left[\frac{M-m}{2}\right]}\frac{1}{k!\left(M-m-2k\right)!}{\left(\frac{a_{x}}{4}\right)}^{k}\sum_{l=0}^{\left[M-m-2k\right]}\frac{\left(M-m-2k\right)!}{l!\left(M-m-2k-l\right)!}{\left(\frac{ik}{2z}\rho_{x}\right)}^{M-m-2k-l}\\ {\left(\frac{1}{2\sigma_{x}^{2}}+\frac{1}{\Lambda^{2}}\right)}^{l}\sqrt{\frac{\pi }{b_{x}}}2^{-\left(M-n+2+l\right)}i^{M-n+2+l}\exp\left(\frac{c_{x}^{2}}{b_{x}}\right){\left(\frac{1}{b_{x}}\right)}^{0.5\left(M-n+2+l\right)}H_{M-n+l}\left(\frac{ic_{x}}{\sqrt{b_{x}}}\right)\\ \sqrt{\frac{\pi }{a_{y}}}\left(m+2\right)!{\left(\frac{1}{a_{y}}\right)}^{m+2}\exp\left[\frac{1}{a_{y}}{\left(\frac{ik}{2z}\rho_{1y}-\frac{\rho_{1y}-\rho_{2y}}{2\Lambda^{2}}\right)}^{2}\right]\\ \sum_{t=0}^{\left[\frac{m+2}{2}\right]}\frac{1}{t!\left(m+2-2t\right)!}{\left(\frac{a_{y}}{4}\right)}^{t}\sum_{l\prime =0}^{\left[m+2-2t\right]}\frac{\left(m+2-2t\right)!}{l\prime !\left(m+2-2t-l\prime \right)!}{\left(\frac{ik}{2z}\rho_{y}\right)}^{m+2-2t-l\prime }{\left(\frac{1}{2\sigma_{y}^{2}}+\frac{1}{\Lambda^{2}}\right)}^{l\prime }\\ \sqrt{\frac{\pi }{b_{y}}}2^{-\left(n+l\prime \right)}i^{n+l\prime }\exp\left(\frac{c_{y}^{2}}{b_{y}}\right){\left(\frac{1}{b_{y}}\right)}^{0.5\left(n+l\prime \right)}H_{n+l\prime }\left(\frac{ic_{y}}{\sqrt{b_{y}}}\right) \end{array}$$

(17) $$\begin{array}{l} I_{9}=\sqrt{\frac{\pi }{a_{x}}}\left(M-m\right)!{\left(\frac{1}{a_{x}}\right)}^{M-m}\exp\left[\frac{1}{a_{x}}{\left(\frac{ik}{2z}\rho_{x}\right)}^{2}\right]\\ \sum_{k=0}^{\left[\frac{M-m}{2}\right]}\frac{1}{k!\left(M-m-2k\right)!}{\left(\frac{a_{x}}{4}\right)}^{k}\sum_{l=0}^{\left[M-m-2k\right]}\frac{\left(M-m-2k\right)!}{l!\left(M-m-2k-l\right)!}{\left(\frac{ik}{2z}\rho_{x}\right)}^{M-m-2k-l}\\ {\left(\frac{1}{2\sigma_{x}^{2}}+\frac{1}{\Lambda^{2}}\right)}^{l}\sqrt{\frac{\pi }{b_{x}}}2^{-\left(M-n+l\right)}i^{M-n+l}\exp\left(\frac{c_{x}^{2}}{b_{x}}\right){\left(\frac{1}{b_{x}}\right)}^{0.5\left(M-n+l\right)}H_{M-n+l}\left(\frac{ic_{x}}{\sqrt{b_{x}}}\right)\\ \sqrt{\frac{\pi }{a_{y}}}\left(m+2\right)!{\left(\frac{1}{a_{y}}\right)}^{m+2}\exp\left[\frac{1}{a_{y}}{\left(\frac{ik}{2z}\rho_{y}\right)}^{2}\right]\\ \sum_{t=0}^{\left[\frac{m+2}{2}\right]}\frac{1}{t!\left(m+2-2t\right)!}{\left(\frac{a_{y}}{4}\right)}^{t}\sum_{l\prime =0}^{\left[m+2-2t\right]}\frac{\left(m+2-2t\right)!}{l\prime !\left(m+2-2t-l\prime \right)!}{\left(\frac{ik}{2z}\rho_{y}\right)}^{m+2-2t-l\prime }{\left(\frac{1}{2\sigma_{y}^{2}}+\frac{1}{\Lambda^{2}}\right)}^{l\prime }\\ \sqrt{\frac{\pi }{b_{y}}}2^{-\left(n+2+l\prime \right)}i^{n+2+l\prime }\exp\left(\frac{c_{y}^{2}}{b_{y}}\right){\left(\frac{1}{b_{y}}\right)}^{0.5\left(n+2+l\prime \right)}H_{n+2+l\prime }\left(\frac{ic_{y}}{\sqrt{b_{y}}}\right) \end{array}$$

with

(18) $$a_{\beta }=\frac{1}{w_{\beta }^{2}}+\frac{1}{2\sigma_{\beta }^{2}}+\frac{1}{\Lambda^{2}}+\frac{ik}{2z}\left(\beta =x,y\right)$$

(19) $$b_{\beta }=\frac{1}{w_{\beta }^{2}}+\frac{1}{2\sigma_{\beta }^{2}}+\frac{1}{\Delta^{2}}-\frac{ik}{2z}-\frac{1}{b_{\beta }}\left(\frac{1}{2\sigma_{\beta }^{2}}+\frac{1}{\Lambda^{2}}\right)$$

(20) $$c_{\beta }=\frac{ik}{2z}\rho_{\beta }-\frac{ik}{2a_{\beta }z}\left(\frac{1}{2\sigma^{2}}+\frac{1}{\Lambda^{2}}\right)\rho_{\beta }$$

By using Equations (8)–(20), the evolution of intensity of PCAAVLB in turbulent biological tissue can be obtained.

## 3. Results and Analyses

In this section, the intensity of a PCAAVLB in turbulent biological tissue is analyzed based on the numerical simulations. In the numerical simulation, the following parameter are chosen, such as λ=0.8 μm, $w_{0x}=w_{0y}=20\;\mu m$, $\sigma_{x}=\sigma_{y}=\sigma =5\;\mu m$, M=1, $S=10^{-4}$, $l_{c}=10\;\mu m$, $l_{0}=1\;\mu m$, and D=4.

**Figure 1:**
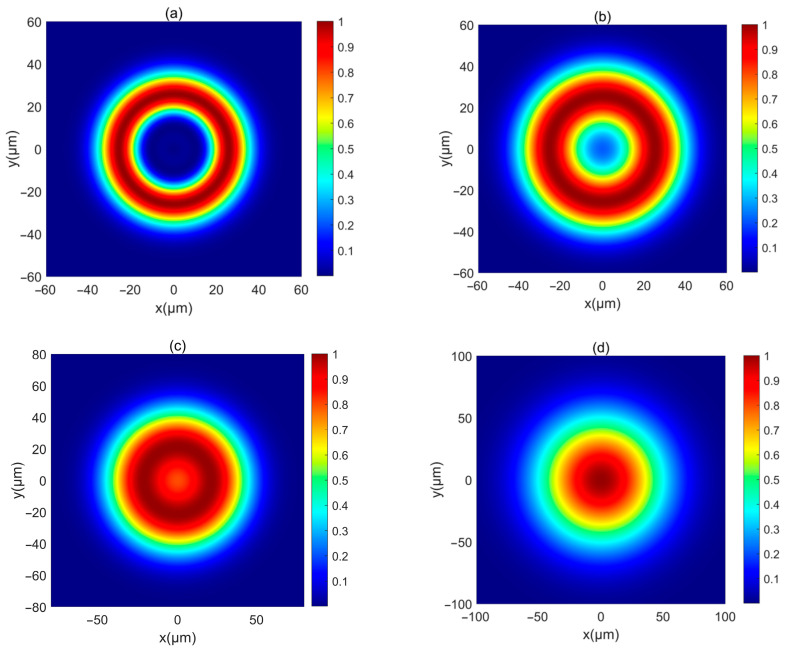
The normalized intensity of a PCAAVLB with a=2, $c_{x}=c_{y}=8$ in free space: (**a**) *z* = 20 μm, (**b**) *z* = 300 μm, (**c**) *z* = 500 μm, and (**d**) *z* = 1000 μm.

**Figure 2:**
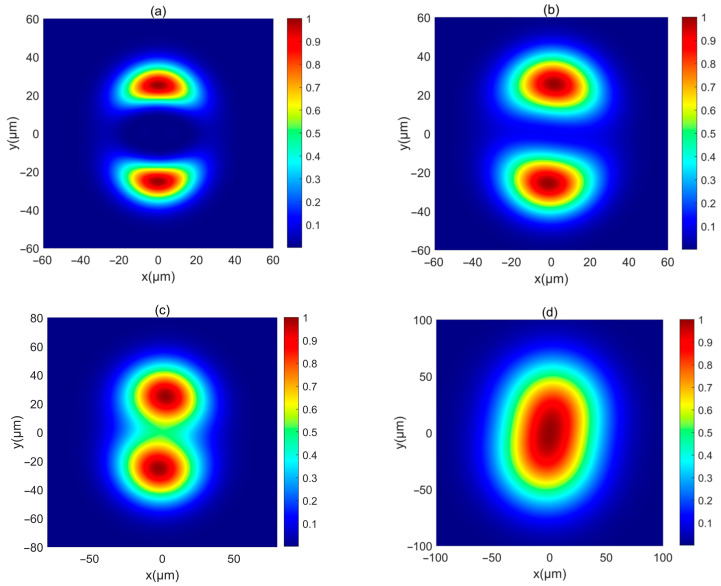
The normalized intensity of a PCAAVLB with a=1, $c_{x}=2$, and *c_y_* = 8 in free space: (**a**) *z* = 20 μm, (**b**) *z* = 300 μm, (**c**) *z* = 500 μm, and (**d**) *z* = 1000 μm.

**Figure 3:**
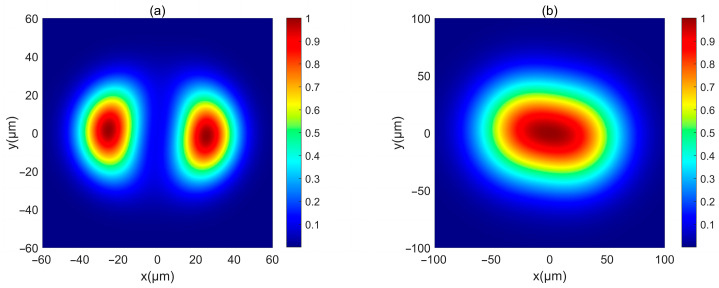
The normalized intensity of a PCAAVLB with a=1 and $c_{x}=8$, and *c_y_* = 2 in free space: (**a**) *z* = 300 μm, and (**b**) *z* = 1000 μm.

First, the intensity of a PCAAVLB with a=2, $c_{x}=c_{y}=8$, and $\sigma_{x}=\sigma_{y}=5\;\mu m$ in free space (Λ=inf) is illustrated in Figure 1. The intensity of this PCAAVLB in free space shows a ring profile at z=20 μm (Figure 1a). As z increases, the hollow width of ring shape of such a PCAAVLB will gradually lessen (Figure 1b) and disappear (Figure 1c). At last, the intensity of this PCAAVLB will become a spot pattern with a Gaussian-like profile (Figure 1d). Then the adjustable parameters change. Figure 2 shows the intensity of a PCAAVLB with a=1, $c_{x}=2$, and $c_{y}=8$ in free space. When $c_{x}\neq c_{y}$, the intensity of this PCAAVLB can show the two-spot pattern at z=20 μm (Figure 2a). The PCAAVLB with $c_{x}=c_{y}$ has the ring profile (Figure 1a). As z increases, the two-spot pattern of this PCAAVLB will overlap (Figure 2b,c). Lastly, the intensity of this PCAAVLB can become an elliptical profile (Figure 2d) when *c_x_* > *c_y_*; the intensity of PCAAVLB with a=1, $c_{x}=8$, and $c_{y}=2$ in free space is shown in Figure 3. The intensity of such a PCAAVLB will have two spots along x-axis (Figure 3a), while the PCAAVLB with *c_x_* < *c_y_* shows two spots along the y-axis (Figure 2a). And this PCAAVLB will evolve into an elliptical beam along the x-axis (Figure 3b). Therefore, the intensity of such PCAAVLB can be controlled by adjustable parameters and the circular or elliptical intensity shape can be obtained by setting the different parameters.

**Figure 4:**
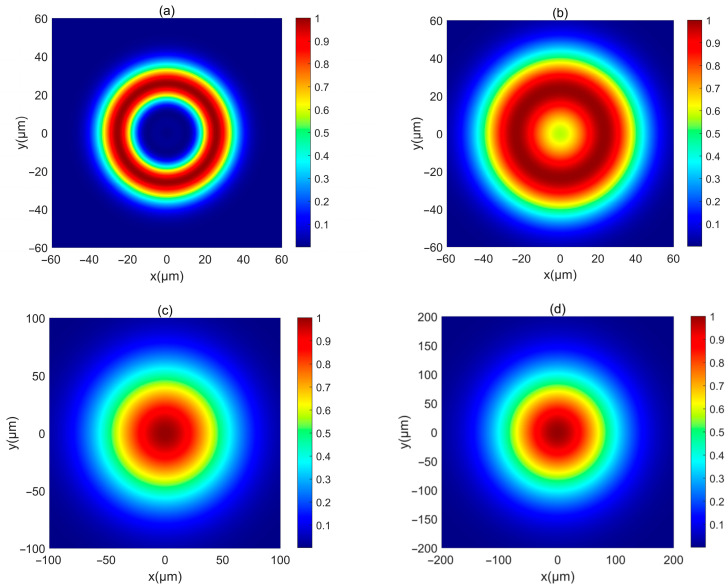
The normalized intensity of a PCAAVLB with a=2 and $c_{x}=c_{y}=8$ in biological tissue for the different *δ*: (**a**) *z* = 20 μm, (**b**) *z* = 150 μm, (**c**) *z* = 300 μm, and (**d**) *z* = 500 μm.

**Figure 5:**
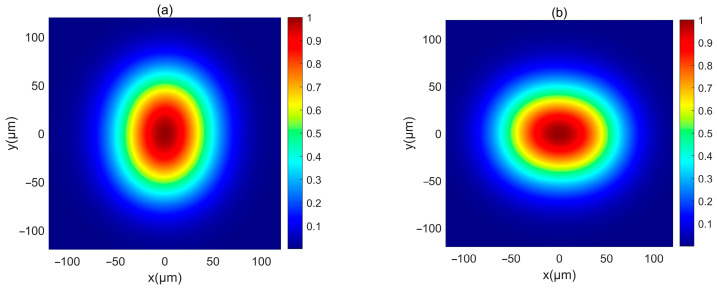
Cross sections of a PCAAVLB with a=−2 and $c_{x}=c_{y}=8$ in biological tissue at *z* = 300 μm: (**a**) *a* = 1, *c_x_* = 2, and *c_y_* = 8; (**b**) *a* = 1, *c_x_* = 8, and *c_y_* = 2.

Next, the intensity of such PCAAVLB in turbulent biological tissue is studied. Figure 4 illustrates the intensity of a PCAAVLB with *a* = 2 and *c_x_* = *c_y_* = 8 in turbulent biological tissue. Intensity shape of this PCAAVLB in turbulent biological tissue at *z* = 20 μm retains its ring shape (Figure 4a), and the hollow center will gradually disappear at *z* = 150 μm (Figure 4b), while the PCAAVLB with *δ* = 5 μm in free space at *z* = 300 μm just has the ring shape (Figure 1a). Therefore, the biological tissue will accelerate the speed of the PCAAVLB ring shape loss. As z increases, this PCAAVLB in turbulent biological tissue will show a Gaussian-like shape (Figure 4c), and the spot of intensity will spread farther (Figure 4d). When *c_x_* ≠ *c_y_*, the intensity of PCAAVLB with *c_x_* < *c_y_* in turbulent biological tissue becomes an elliptical profile with the long axis along the y-axis at *z* = 300 μm (Figure 5a), while the same PCAAVLB in free space will evolve into a two-spot pattern (Figure 2c). In addition, the intensity of PCAAVLB with *c_x_* > *c_y_* in turbulent biological tissue will evolve into an elliptical Gaussian profile with the long axis along the x-axis at *z* = 300 μm (Figure 5b). From Figure 4 and Figure 5, one concludes that the PCAAVLB in turbulent biological tissue will evolve into one spot pattern faster as z increases.

**Figure 6:**
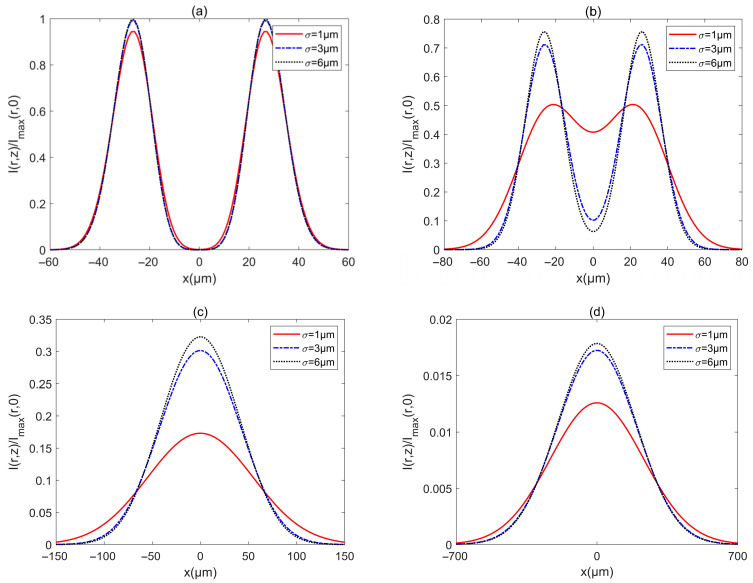
Cross sections of intensity of a PCAAVLB with a=−4 and $c_{x}=c_{y}=8$ in biological tissue for the different *δ*: (**a**) *z* = 20 μm, (**b**) *z* = 100 μm, (**c**) *z* = 300 μm, and (**d**) *z* = 1000 μm.

To see the beam parameters on the intensity of a PCAAVLB in turbulent biological tissue, Figure 6 shows the intensity $I\left(r,z\right)/I_{\max}\left(r,0\right)$ of a PCAAVLB with a=−4, $c_{x}=c_{y}=8$, and M=2 in biological tissue for the different σ. The intensity of PCAAVLBs with the different σ in turbulent biological tissue has the same profile at z=20 μm (Figure 6a). As z increases, such PCAAVLB with smaller σ in turbulent biological tissue will first lose the ring profile (Figure 6a) and gradually become a Gaussian-like profile (Figure 6c). As z increases further, such PCAAVLBs with the different σ can all have Gaussian-like patterns, such PCAAVLBs with smaller σ will have a larger width, and the intensity of PCAAVLB with smaller σ will decrease faster (Figure 6d). Thus, the speed of evolution can be controlled by σ, and the PCAAVLBs with a smaller σ will evolve into a Gaussian shape faster and have a smaller intensity.

**Figure 7:**
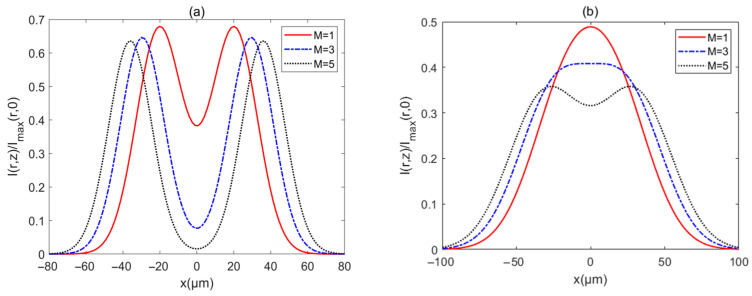

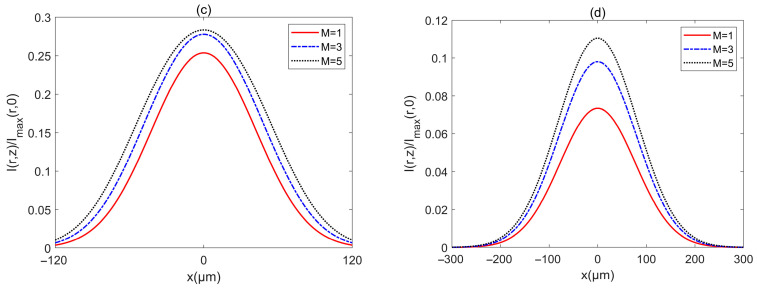
Cross sections of intensity of a PCAAVLB with a=−4 and $c_{x}=c_{y}=8$ in biological tissue for the different *M*: (**a**) *z* = 100 μm, (**b**) *z* = 200 μm, (**c**) *z* = 300 μm, and (**d**) *z* = 500 μm.

The intensity $I\left(r,z\right)/I_{\max}\left(r,0\right)$ of a PCAAVLB with a=−4, $c_{x}=c_{y}=8$, and σ=2 μm in turbulent biological tissue for the different M is illustrated in Figure 7. The PCAAVLB with a larger M has a bigger width at z=100 μm (Figure 7a). As z increases, the PCAAVLB with a smaller M in turbulent biological tissue will first lose its ring profile, while the intensity of the PCAAVLB with a larger M will have a lower intensity (Figure 7b). As z increases further, the PCAAVLB with a different M can gradually become a Gaussian profile (Figure 7c), and such a PCAAVLB with a smaller M in turbulent biological tissue will have a larger intensity although all PCAAVLBs have the Gaussian profile (Figure 7d).

**Figure 8:**
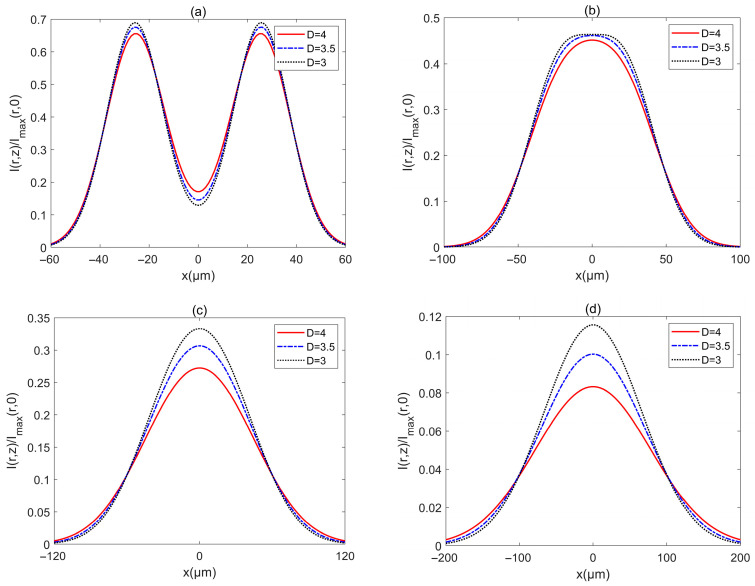
Cross sections of intensity of a PCAAVLB with a=−4 and $c_{x}=c_{y}=8$ in biological tissue for the different *D*: (**a**) *z* = 100 μm, (**b**) *z* = 200 μm, (**c**) *z* = 300 μm, and (**d**) *z* = 500 μm.

**Figure 9:**
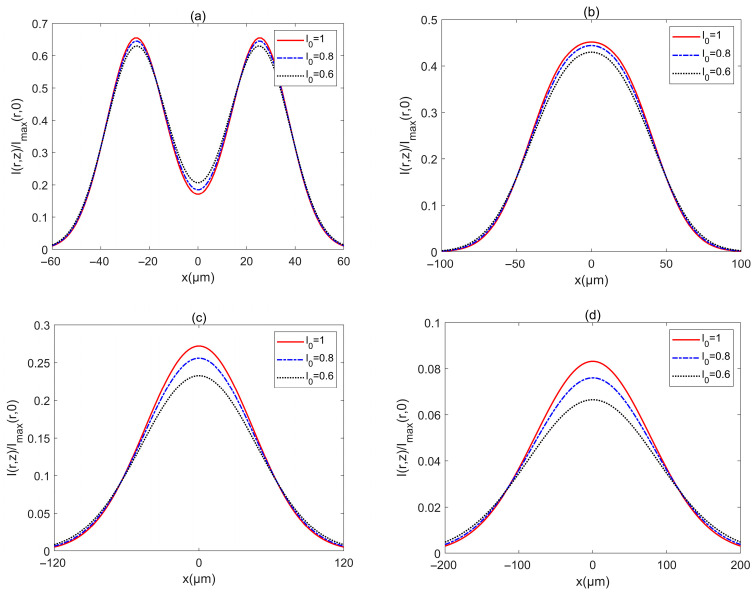
Cross sections of intensity of a PCAAVLB with a=−4 and $c_{x}=c_{y}=8$ in biological tissue for the different $l_{0}$: (**a**) *z* = 100 μm, (**b**) *z* = 200 μm, (**c**) *z* = 300 μm, and (**d**) *z* = 500 μm.

The influences of parameters of turbulent biological tissue on the intensity of PCAAVLB are shown in Figure 8 and Figure 9. The intensity $I\left(r,z\right)/I_{\max}\left(r,0\right)$ of a PCAAVLB with a=−4, $c_{x}=c_{y}=8$, and σ=2 μm in turbulent biological tissue for the different D is illustrated in Figure 8. The intensity profile of such a PCAAVLB in turbulent biological tissue with different D remains the ring profile at z=100 μm (Figure 8a). As z increases, such PCAAVLB in a turbulent biological tissue with a larger D will first lose its ring shape (Figure 8b), evolve into a Gaussian profile, and the beam in a turbulent biological tissue with a larger D will have a larger intensity. (Figure 8c). The PCAAVLB in a turbulent biological tissue with a larger D will have a larger spot at z=500 μm and remain the larger intensity (Figure 8d).

Figure 9 shows the intensity $I\left(r,z\right)/I_{\max}\left(r,0\right)$ of a PCAAVLB with a=−4, $c_{x}=c_{y}=8$, and σ=2 μm in turbulent biological tissue for different *l*_0_. The intensity profile of such a PCAAVLB in turbulent biological tissue with different *l*_0_ remains almost similar at *z* = 100 x3BCm (Figure 9a). As z increases, the PCAAVLB in turbulent biological tissue for the different *l*_0_ will have similar evolution properties (Figure 9b,c). At last, the PCAAVLB in turbulent biological tissue with different *l*_0_ will have a similar spot and the beam with larger *l*_0_ will have a larger intensity. Therefore, one can conclude that the PCAAVLB in turbulent biological tissue with a larger *D* or a smaller *l*_0_ will evolve from a ring profile into a Gaussian-like profile faster, and such a PCAAVLB in turbulent biological tissue with a larger *D* or a smaller *l*_0_ will have a larger intensity.

## 4. Conclusions

Vortices are ubiquitous in nature, such as in fluids, smoke rings, and tornados, and vortices have also been observed in light. In this work, a new beam carried vortex, which is named PCAAVLB, is introduced, and the intensity of such PCAAVLB in turbulent biological tissue is derived. The intensity shape of such PCAAVLB in free space can have the ring shape or a two-spot pattern, and the intensity profile is controlled by adjustable initial parameters. The intensity of such a PCAAVLB can gradually evolve into a Gaussian-like profile. And the intensity of such a PCAAVLB with a smaller M or σ in turbulent biological tissue will lose its ring shape faster, and the intensity profile of such a PCAAVLB with smaller σ in turbulent biological tissue will have a larger spot, while the PCAAVLB with different M in turbulent biological tissue has almost the same intensity profile at a larger distance. In addition, the PCAAVLB in turbulent biological tissue with larger D or smaller $l_{0}$ will evolve from a ring profile into a Gaussian-like profile faster and have a larger intensity. The obtained results may have potential applications in laser sensing under biological tissue environments and laser imaging in biology.

## Data Availability

The data presented in this work are available upon request from the corresponding author.
